# Case Report: Difficult radial artery sheath removal: a report of two cases and review of the literature

**DOI:** 10.3389/fcvm.2025.1581346

**Published:** 2025-05-02

**Authors:** Bangguo Yang, Mengqi Yeh, Jie Bai

**Affiliations:** ^1^ Department of Cardiology, Fuwai Yunnan Hospital, Chinese Academy of Medical Sciences, Kunming, China; ^2^ Department of Cardiology, Affiliated Cardiovascular Hospital of Kunming Medical University, Kunming, China

**Keywords:** coronary artery disease, coronary angiography, transradial, radial artery spasm, sheath removal

## Abstract

The transradial approach to coronary angiography and interventional therapy has gained worldwide popularity in recent years due to its lower rate of vascular complications compared to the femoral approach. We present two cases of patients who experienced difficulties in removing the radial artery sheath. In the first case, the sheath inadvertently fractured within the radial artery, but it was successfully extracted using hemostatic forceps. In the second case, a severe spasm of the radial artery complicated sheath removal, resulting in the sheath becoming stretched and deformed. Ultimately, the deformed sheath was removed slowly and successfully with the assistance of a guidewire and catheter. Furthermore, we conducted a review of existing literature to delineate management strategies for situations where sheath removal poses challenges.

## Introduction

Coronary interventional therapy via the radial artery approach (1) has been extensively utilized since its initial application in 1989 and has progressively been adopted for peripheral vascular interventions (2) and neuroendovascular treatments (3, 4). However, complications related to the radial artery are also on the rise, including radial artery spasm, perforation, and occlusion (5). In some instances, severe spasm may lead to radial artery transection (6), catheter or sheath entrapment (7), or even sheath rupture (8). In this report, we present two cases involving challenging sheath removal procedures. The first case describes an incident in which the sheath inadvertently broke within the radial artery, while the second case details severe deformation of the sheath resulting from significant radial artery spasm. Additionally, we review the existing literature to delineate management strategies for patients encountering challenges with radial artery sheath removal.

## Case presentation

The first patient is a 68-year-old female who was admitted to the hospital due to intermittent chest and back pain that had persisted for 4 years, with a recent recurrence over the past week. Her medical history included a previous episode of acute anterior wall myocardial infarction in 2019, for which she underwent stent implantation at an external hospital via the right radial artery and was compliant with her medication regimen. Echocardiography revealed a left ventricular ejection fraction of 62% with mild mitral valve regurgitation. Following a comprehensive examination, no significant abnormalities were identified. On February 2, 2024, the patient underwent an invasive coronary angiography (CA). During the procedure, the right radial artery was successfully punctured under local anesthesia, and a 6-F sheath (Terumo, Tokyo, Japan) was inserted. Heparin (3,000 units) and nitroglycerin (100 µg) were given through the sheath. Subsequently, a 5-F TIG diagnostic catheter (Terumo, Tokyo, Japan) was advanced over a 0.035-inch J-tipped guidewire (Cordis) into the ascending aorta to complete the selective angiography examination of the left and right coronary arteries. The procedure was performed smoothly, and the patient did not report any discomfort during the operation. The CA demonstrated that the original stent in the proximal left anterior descending artery (LAD) was not obstructed, while a plaque was observed in the middle segment of the right coronary artery (RCA). It was recommended that the patient continue with medical treatment, and preparations were made to remove the arterial sheath. However, upon attempting to remove the sheath, it was discovered that the fractured catheter fragment was confirmed to be entirely intravascular, with no visible stump protruding from the skin. Immediate compression of the puncture site was applied in order to prevent blood loss.

**Figure 1:**
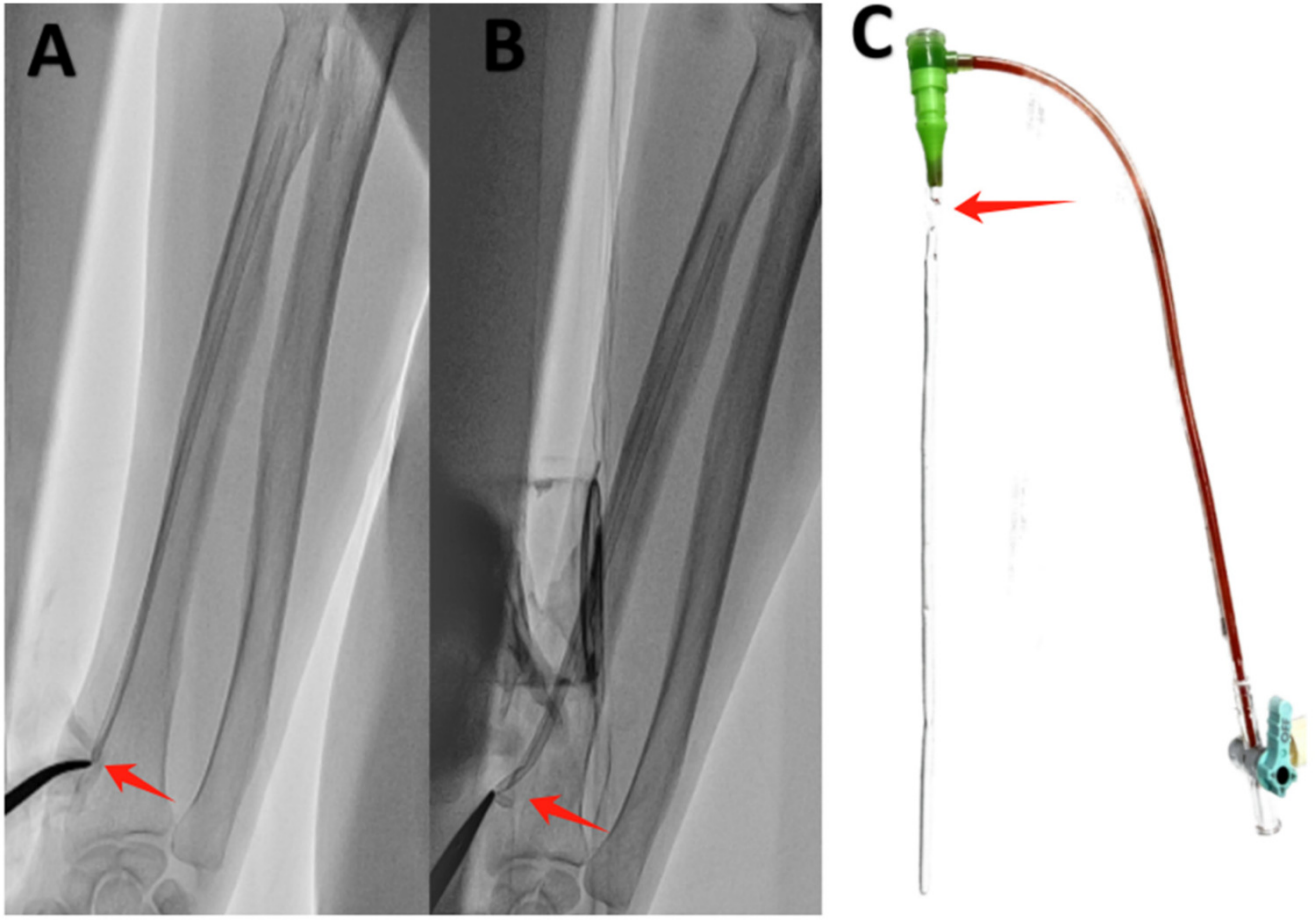
Sheath rupture inside the radial artery. **(A)** The sheath stump was situated below the puncture port (red arrow). **(B)** The sheath stump was grasped with hemostatic forceps (red arrow). **(C)** The broken 6-F sheath (Terumo, Tokyo, Japan) (red arrow).

The hemostat was initially positioned at the radial artery puncture port with the objective of identifying the sheath stump under radiofluoroscopy and determining its distance from the puncture site. Fortunately, the sheath stump was identified in a position just below the puncture port (Figure 1A), thereby allowing for successful removal with the hemostat. It was essential to exercise extreme care and patience during the procedure to prevent any damage to the radial artery or potential sheath breakage, which would have necessitated surgical intervention. After multiple attempts, the sheath stump was finally grasped after widening the puncture port with a blade and inserting the hemostatic forceps into the subcutaneous tissue under the guidance of fluoroscopy (Figure 1B). Subsequently, the sheath was carefully and gently removed without encountering any significant resistance. Following the removal of the sheath, immediate pressure application and bandaging with a radial artery compressor were performed. The spliced sheath sections were examined to ensure that no foreign matter remained within the body (Figure 1C). The patient reported no discernible discomfort during or following the extraction. Four hours post-operatively and on the following day, the pulse in the right radial artery was observed to be robust, indicating the absence of complications.

**Figure 2:**
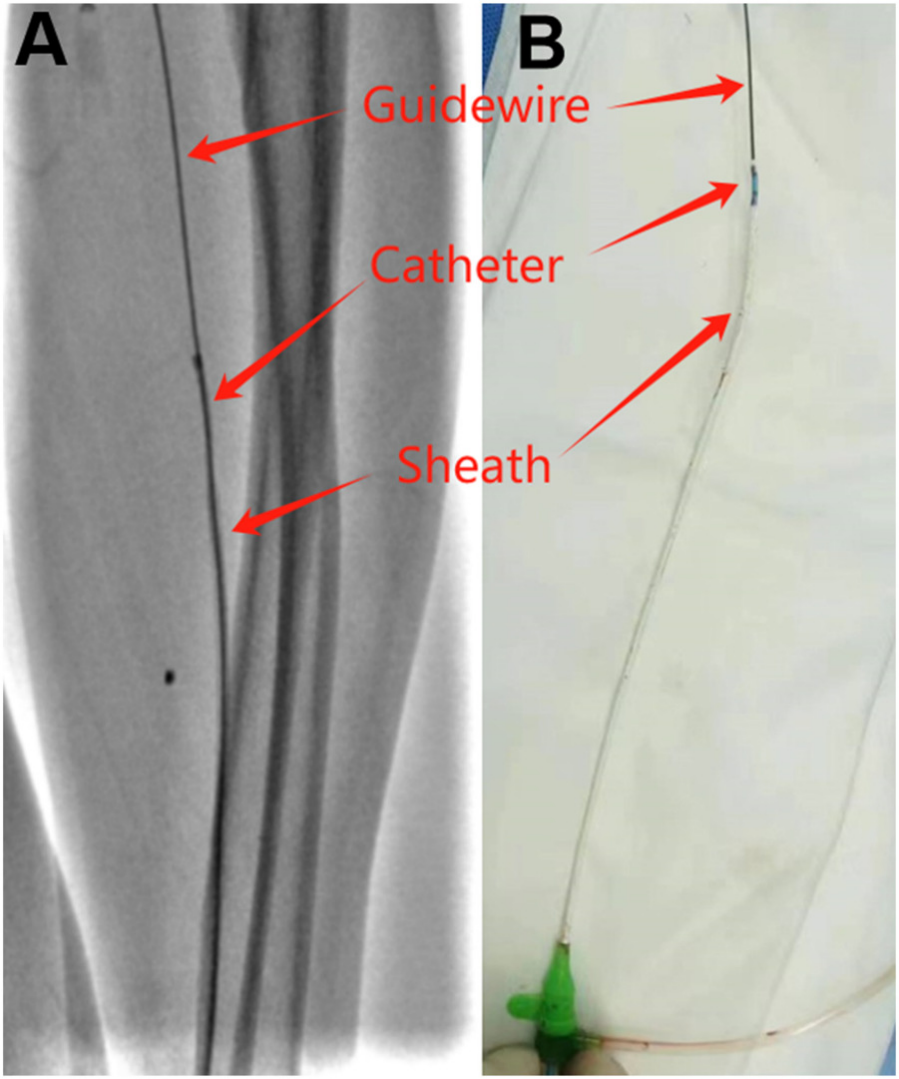
Deformed sheath removal with guidewire and catheter assistance. **(A)** Guidewire, catheter and sheath under fluoroscopy. **(B)** Guidewire, catheter and sheath *in vitro*.

The second patient is a 57-year-old male with a history of myocardial infarction and coronary stent implantation via his right radial artery. He was recently admitted to the hospital due to recurrent chest pain following physical activity. Due to occlusion of the right radial artery, it was decided to perform coronary angiography via the left radial artery. After successful puncture, a 6F Terumo radial artery sheath was inserted, and 3,000 units of heparin along with 100 µg of nitroglycerin were administered through the sheath. Coronary angiography was subsequently completed using 5F JL3.5 and JR 3.5 catheters, revealing occlusion of the posterior descending artery (PDA). A stent was successfully implanted in the RCA-PDA, and the procedure proceeded smoothly, with no reported severe discomfort during the operation. However, upon removal of the 6F Terumo radial artery sheath at the conclusion of the operation, the radial artery experienced spasm, complicating the removal process and resulting in deformation of the sheath. When the sheath deformed, we promptly halted the operation and administered nitroglycerin through the sheath. We then attempted to advance Terumo's own puncture guidewire (0.021 inch), but this was unsuccessful due to the guidewire's excessive softness. Subsequently, we employed a 0.035-inch J-tipped guidewire, which allowed us to successfully advance the guidewire into the brachial artery. With the guidewire's support, we were able to maneuver the 5F JR3.5 catheter into the sheath, achieving integration of the guidewire, contrast catheter, and sheath (Figure 2). Finally, with the assistance of the guidewire and catheter, we carefully and successfully removed the deformed sheath. Post-operatively, the patient reported no significant forearm pain, and the radial artery remained patent both immediately after the procedure and the following day.

## Discussion

In this article, we described two cases of patients who suffered different difficulties during sheath removal. The fractured sheath was successfully extracted using hemostatic forceps in one case, and in the other case, a 0.035-inch guidewire and catheter together were used to cannulate the deformed sheath, which was then withdrawn from the severely spastic radial artery and removed uneventfully. The cause of the sheath fracture in the first case remains unclear; however, radial artery spasm is the most likely etiology. Therefore, during coronary angiography, preventing spasm is crucial, and operators should consider using smaller-sized sheaths, as the sheath-to-artery ratio is an important factor in sheath size selection. In the case of this female patient, utilizing a 5 French or even a 4 French sheath for diagnostic angiography can help mitigate the risk of sheath rupture. Radial artery spasm, as observed in the second case, is a relatively common complication in interventional cardiology, with an incidence ranging from 6.8% to 30% (9). However, not all sheath removals occur without complications; if not managed appropriately, sheath removal can increase patient discomfort and may lead to sheath rupture or radial artery injury. Our percutaneous approach in both cases facilitated the minimally invasive retrieval of the radial artery catheter without the need for surgical intervention. Although straightforward, these techniques can be beneficial in similar circumstances.

In Table 1, we summarized eight case reports of sheath-related complications that occurred during transradial artery interventions, involving five females and three males (6–8, 10–14). The ages of the patients ranged from 42 to 78 years. Among the eight patients, six underwent coronary angiography via the radial artery, one underwent cerebral angiography via the radial artery (6), and one underwent a peripheral angiogram via the radial artery (7). Regarding the entry route and sheath diameter, seven patients had right radial artery access (comprising six 6F sheaths and one 5F sheath), while one patient (7) had left radial artery access (6F sheath). In terms of complications, six patients experienced severe radial artery spasm, one patient had a fractured sheath inside the artery (8), and one patient developed a chronic infection that is secondary to a retained macroscopic fragment of hydrophilic radial artery sheath (13). Various methods were employed for the removal of residual sheaths, including removal with force (one case), Infusion of ViperSlide**™** or Rotaglide**™** lubricant (three cases), surgical intervention (three cases), and brachial plexus block (one case).

**Table 1:** Review of sheath-related complications that occurred during transradial artery access.

No.	Author	Year	Age	Sex	Indication	Approach	Sheath name	Size	Complication	Treatment strategy	Outcome
1	Olechowski (8)	2013	42	F	CA	Right RA	23 cm COOK flexor radial sheath	6Fr	Fractured sheath	Vascular surgery	Tie off the radial artery; full recovery
2	Repanas (10)	2015	53	M	CA	Right RA	Jacky 6Fr catheter (Terumo Medical Corporation, Somerset, New Jersey)	6Fr	Severe RA spasm	Deliver ViperSlide™ (Cardiovascular Systems, Inc. St. Paul, Minnesota)	Without complications
3	Fidone (11)	2018	68	M	ca	Right RA	5Fr hydrophilic sheath (Terumo Medical Corporation)	5Fr	Severe RA spasm	Inject ViperSlide lubricant	Recovered well
4	Raje (12)	2018	52	M	Cardiac catheterization	Right RA	Glidesheath Slender; Jacky 5Fr catheter (Terumo Medical Corporation, Tokyo, Japan)	6Fr	Severe RA spasm	Infusion Rotaglide™ (Boston Scientific, Marlborough, MA)	Without complications
5	Ghazala (13)	2019	62	F	CA	Right RA	Introducer sheatTerumo Europe, Belgium)	6Fr	Chronic infection	3 surgical interventions	The wound had healed without infection
6	Nazir (7)	2020	69	F	Peripheral angiogram	Left RA	Terumo 6Fr R2P destination slender 119 cm sheath	6Fr	Severe RA spasm	Sheath lost its structural integrity and fragmented requiring surgical removal	Recovered well
7	Kovacs (14)	2022	78	F	CA	Right RA	6Fr × 65 cm sheath	6Fr	Severe RA spasm	Brachial Plexus Block	Without complications
8	Kurauchi (6)	2024	60	F	Neuroendovascular therapy	Right RA	Axcelguide (Medikit)	6Fr	Difficult removal	Pulled out the Axcelguide with force	Dislodged blood vessel; subcutaneous hematoma and pain

CA, coronary angiogram; RA, radial artery; Fr, French.

Difficulties in sheath removal are encountered not only with the radial artery approach but become particularly pronounced when employing long sheaths (15). Furthermore, comparable challenges may manifest during interventions involving other peripheral vessels. In a previous study (16), a 31-year-old patient underwent an electrophysiology procedure via the right femoral vein. During the procedure, the 7F sheath fractured within the femoral vein. Consequently, A small venotomy was performed and surgeons extracted the segment of the sheath in the femoral vein successfully. In a separate patient undergoing a cerebral angiogram through right common femoral artery, the dislodged 6F sheath tip was confirmed to be entirely intravascular (17). After the Amplatz Goose Neck Snare could not retract it, the dislodged sheath tip was successfully removed with balloon assisted. Furthermore, prior studies have indicated that arterial cannulas frequently malfunction and can become lodged in the radial artery (18–20). Arterial lines are frequently indicated and routinely used in the care of perioperative and critically ill patients (21). Once compromised, the removal of these catheters can be challenging. In previously reported cases, surgical intervention was the predominant method for removal, while in three instances (22, 23), ultrasound-guided percutaneous retrieval of transected arterial lines was the useful approach. The studies mentioned above illustrate various techniques for sheath or severed end removal, thereby enhancing our clinical practice knowledge. Similarly, our cases present two effective solutions.

When encountering difficulties in sheath removal, it is essential to consider not only the possibility of spasm but also to evaluate additional factors such as the influence of hydrophilic coating, sheath shaft thickness, and the material properties of different sheath types. A systematic review and meta-analysis demonstrated that hydrophilic-coated sheaths significantly reduce both radial artery spasm and periprocedural pain or discomfort compared to non-coated sheaths, with no significant effect on radial artery occlusion, hematoma, or pseudoaneurysm (24).

**Figure 3:**
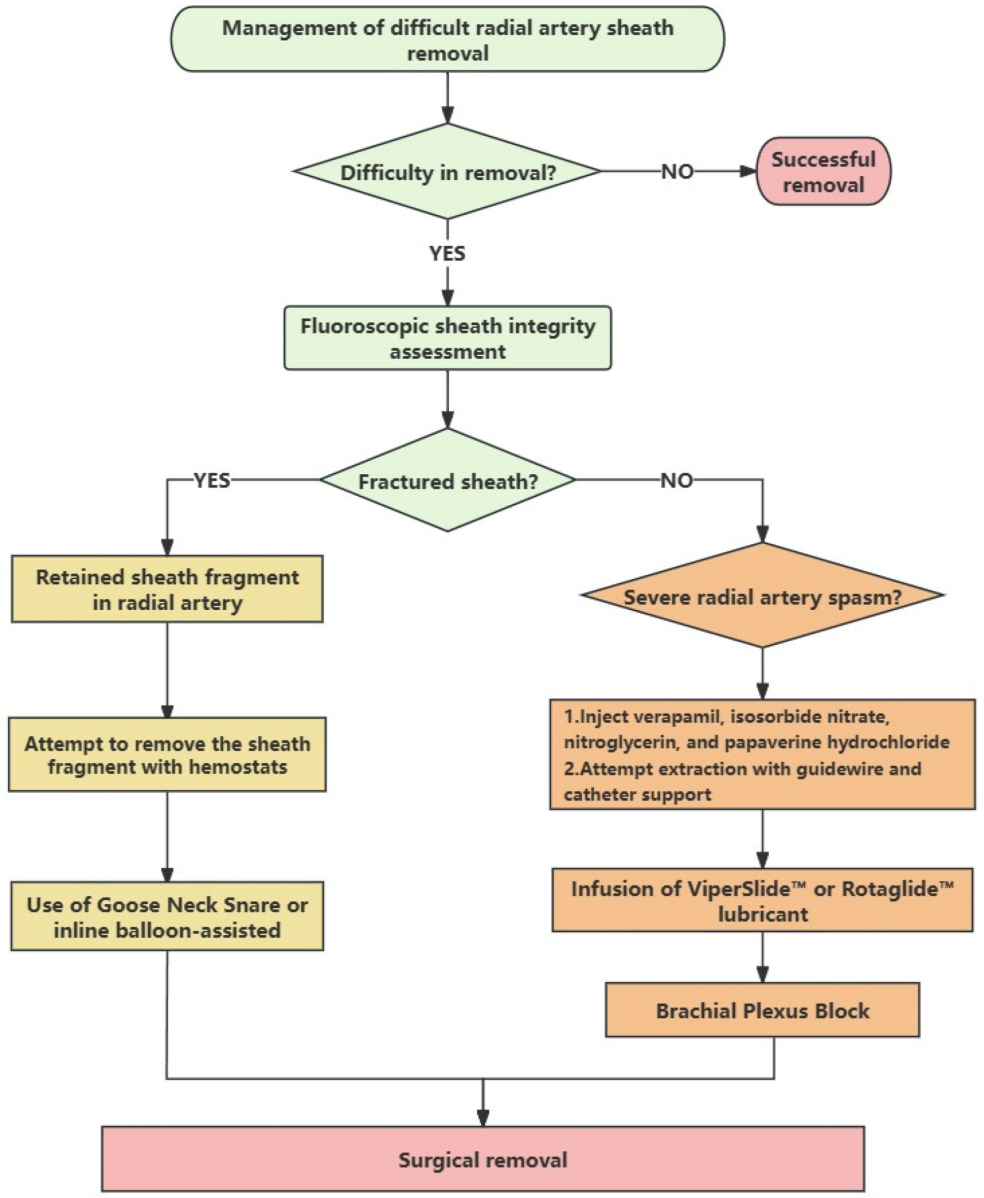
Management of difficult radial artery sheath removal.

Figure 3 presents a clinical algorithm outlining treatment strategies for challenging radial artery sheath removal, designed to provide a practical framework for clinicians. This protocol was developed based on retrospective case analyses, though its application should always be tailored to individual patient circumstances. Clinicians may employ single or combined strategies to ensure safe sheath extraction while preventing serious complications. Additionally, for cases of refractory vasospasm, Papaverine may be considered as an effective vasodilator—an option frequently utilized by vascular and cardiac surgeons in clinical practice (25, 26). Finally, it should be noted that such complications are relatively uncommon in clinical practice. To date, the exact incidence rate remains unclear. To obtain more accurate epidemiological data, it is warranted to establish a more comprehensive database for systematic analysis.

## Conclusions

In the context of sheath-related complications, prevention is far easier than treatment. When encountering difficulty in removing the sheath, it is essential to quickly ascertain the underlying cause and to avoid using aggressive techniques, as these may result in severe complications. Once complications occur, it is imperative to implement a variety of treatment strategies to ensure the safe and effective removal of the sheath.

## Data Availability

The original contributions presented in the study are included in the article/Supplementary Material, further inquiries can be directed to the corresponding author.

## References

[1] Krittanawong C Uppalapati L Virk HUH Qadeer YK Irshad U Wang Z Complications of radial vs femoral access for coronary angiography and intervention: what do the data tell US? Am J Med. (2024) 137(6):483–9. 10.1016/j.amjmed.2024.02.022 38387541

[2] Song C Carlson SJ. Radial artery access for peripheral vascular interventions: a review of the literature. Ann Vasc Surg. (2024) 107:55–9. 10.1016/j.avsg.2023.11.058 38582199

[3] Rentiya ZS Kuhn AL Hutnik R Shazeeb MS De Leacy RA Goldman D Transradial access for cerebral angiography and neurointerventional procedures: a meta-analysis and systematic review. Interv Neuroradiol. (2024) 30(3):404–11. 10.1177/15910199221112200 35837726 PMC11310734

[4] Joshi KC Beer-Furlan A Crowley RW Chen M Munich SA. Transradial approach for neurointerventions: a systematic review of the literature. J Neurointerv Surg. (2020) 12(9):886–92. 10.1136/neurintsurg-2019-015764 32152185 PMC7476364

[5] Roy S Kabach M Patel DB Guzman LA Jovin IS. Radial artery access complications: prevention, diagnosis and management. Cardiovasc Revasc Med. (2022) 40:163–71. 10.1016/j.carrev.2021.12.007 34952824

[6] Kurauchi Y Onda T Takahashi K Inamura S Daibou M Nonaka T. Difficult 6F guiding sheath removal using the transradial artery approach: a case report. J Neuroendovasc Ther. (2024) 18(8):224–9. 10.5797/jnet.cr.2024-0026 39166096 PMC11333153

[7] Nazir S Nesheiwat Z Syed MA Gupta R. Severe radial artery spasm causing entrapment of the terumo radial to peripheral destination slender sheath: a case report. Eur Heart J Case Rep. (2020) 4(2):1–4. 10.1093/ehjcr/ytaa038 32352072 PMC7180690

[8] Olechowski B Purkiss M Strike P. Radial artery damage due to sheath fracture: unpredicted complication. Heart. (2013) 99(5):353–4. 10.1136/heartjnl-2012-302825 23175128

[9] Kristić I Lukenda J. Radial artery spasm during transradial coronary procedures. J Invasive Cardiol. (2011) 23(12):527–31. 22147403

[10] Repanas T Christopoulos G Brilakis ES. Administration of ViperSlide™ for treating severe radial artery spasm: case report and systematic review of the literature. Cardiovasc Revasc Med. (2015) 16(4):243–5. 10.1016/j.carrev.2015.02.008 25800340

[11] Fidone E Price J Gupta R. Use of ViperSlide lubricant to extract entrapped sheath after severe radial artery spasm during coronary angiography. Tex Heart Inst J. (2018) 45(3):186–7. 10.14503/THIJ-17-6394 30072861 PMC6059514

[12] Raje V Christopher S Hopkinson DA Kania DA Jovin IS. Administration of rotaglide™ solution for treating refractory severe radial artery spasm: a case report. Cardiovasc Revasc Med. (2018) 19(8S):56–7. 10.1016/j.carrev.2018.05.001 29779974

[13] Ghazala CG Marrow BA Kearney D Harrison JWK. Infected chronic sinus secondary to a retained fragment of radial artery introducer sheath following percutaneous coronary intervention (PCI). BMJ Case Rep. (2019) 12(3):e227136. 10.1136/bcr-2018-227136 30862671 PMC6441256

[14] Kovacs PL Deutch Z Castillo D. Brachial plexus block for removal of retained radial artery sheath. Cureus. (2022) 14(12):e33068. 10.7759/cureus.33068 36726880 PMC9886372

[15] Vadalà G Sucato V Costa F Castriota F Nerla R Roscitano G Uncommon carotid artery stenting complications: a series by images. J Pers Med. (2024) 14(3):250. 10.3390/jpm14030250 38540992 PMC10971077

[16] Pillarisetti J Biria M Balda A Reddy N Berenbom L Lakkireddy D. Integrity of vascular access: the story of a broken sheath!. J Vasc Nurs. (2009) 27(3):75–7. 10.1016/j.jvn.2009.07.001 19699446

[17] Jamshidi N Chiang J. Inline balloon-assisted vascular sheath fragment removal. CVIR Endovasc. (2020) 3(1):53. 10.1186/s42155-020-00142-1 32886241 PMC7474008

[18] Moon SK Gong JC Kim JH Lee KC Kim HY Choi EK A retained catheter fragment in radial artery caused by accidental catheter transection during arterial catheter removal. J Anesth. (2012) 26(4):625–6. 10.1007/s00540-012-1388-4 22484914

[19] Nielsen JH Thomsen AB Thomsen NO. An unnoticed retained cannula fragment in the radial artery: should ultrasound investigation be included in guidelines? Eur J Anaesthesiol. (2014) 31(2):118–20. 10.1097/EJA.0b013e328361a5a6 23635995

[20] Tollinche L Jackson J La M Desiderio D Yeoh C. Case report: transection of radial arterial catheter requiring surgical intervention. J Intensive Crit Care. (2018) 4(1):3. 29780973 PMC5954833

[21] Imbrìaco G Monesi A Spencer TR. Preventing radial arterial catheter failure in critical care—factoring updated clinical strategies and techniques. Anaesth Crit Care Pain Med. (2022) 41(4):101096. 10.1016/j.accpm.2022.101096 35490863

[22] Derycke L Mallios A. Ultrasound-guided percutaneous retrieval of transected radial artery catheter. J Vasc Access. (2021) 22(1):151–3. 10.1177/1129729819899807 31928296

[23] Alaeddin H Elsaadany A Rashid Akhtar M. Ultrasound-guided percutaneous retrieval of non-radiopaque radial line using a microsnare. CVIR Endovasc. (2023) 6(1):59. 10.1186/s42155-023-00407-5 (published correction appears in CVIR Endovasc. 2024 7(1):3. doi: 10.1186/s42155-023-00419-1). 38019316 PMC10686901

[24] Hukamdad M Adachi K Soliman Y Ezzeldin R Tatapudi SVV Ezzeldin M. Hydrophilic-coated sheaths for reducing radial artery spasm during transradial procedures: a systematic review and meta-analysis. Interv Neuroradiol. (2025). 10.1177/15910199251329150 40140363 PMC11951130

[25] Ashrafi S Alam S Sultana A Raj A Emon NU Richi FT Papaverine: a miraculous alkaloid from opium and its multimedicinal application. Molecules. (2023) 28(7):3149. 10.3390/molecules28073149 37049912 PMC10095881

[26] Tanaka-Totoribe N Nakamura E Kuwabara M Onizuka S Yamamoto R. Optimal concentration of papaverine for the inhibition of internal thoracic artery vasospasm during coronary artery bypass graft surgery. Braz J Cardiovasc Surg. (2025) 40(1):e20240058. 10.21470/1678-9741-2024-0058 39937755 PMC11816267

